# Summary of Discussions

**Published:** 1886-05

**Authors:** 


### PROCEEDINGS OF SOCIETIES.

### DISCUSSIONS.

### Summary of Discussions at The Michigan State Dental Association, held at Ann Arbor, 1886.

DISCUSSION UPON THE PAPER OF DR. MARTIN.

In answer to an inquiry by Dr. Shattuck, *Dr. Martin* stated that the wisdom teeth were erupted in the boy, but there was no history of any irritation in the eruption of the wisdom teeth; that near where the disease started was a carious tooth, which was extracted three days after the disease began; that was the only cause found for irritation, that the carious tooth was on the right side, and the sinus leading to the swelling was on the left side.

*Dr. Lowe* stated he thought the case was an abscess at the start, and had not been treated well before Dr. Martin commenced to treat it.

*Professor Taft* said, in reference to the beginning of the case, it was simply through inflammation of the periosteum, with rapid extension to the other tissues about it, especially the gum and the masseter muscles; that the muscles were not involved to any considerable extent on the right side, but were largely involved on the left side. That the inflammation extended rapidly until all these tissues were deeply involved. In some organizations, this rapidity of extension would not take place, but there was great susceptibility in this particular case. The large involvement of the tissues on the left side, and the rapidity of the extension of the inflammation were remarkable in this case. Constitutional difficulty no doubt existed and the local disturbance interfered with the general system. The pus was exuding from the gums when he (the speaker) first saw it, and that pus would pass into the alimentary canal with the food, which would produce a phase of poisoning, and would tend to impair the general functions of the system. It may be that the young man was subjected to malarious influences, perhaps to cold. It is a question of great importance to every dental practitioner to know if a tooth is capable of producing such serious results as appeared in this case; in the speaker's opinion, that was not the sole cause of the difficulty, although it may have been a source of irritation. Another important point is the promptness with which the affection yielded to the surgical operation, after the external opening was made. The result showed that to give free drainage was the rational thing to do, as rapid restoration and recovery speedily resulted after that. There was no dead bone at the right side, according to the speaker's judgment, although there might have been. There may have been sequestra on the right side before the patient came there, but not when he came.

*Dr. Shattuck* stated he wished to relate a case from his experience. A colored man came to his office to have a tooth extracted, and it was extracted in the ordinary manner.

Immediately afterwards, the patient, who was a fireman on a steamboat, went on a trip up Lake Superior. The atmosphere of Lake Superior was colder than that of Detroit, and his duties as fireman kept him in different temperatures. That he suffered a great deal of pain on his way up the Lake in his left jaw, which commenced swelling, and he was obliged to quit work when he reached the upper part of the Lake. He returned to Detroit with his face very much swollen; necrosis set in, he lost six of his teeth on that side and the greater portion of the lower maxillary, and he attributed it all to the extraction of the tooth, and threatened a suit for malpractice, which, however, was subsequently abandoned. The speaker at that time wished for just such a case as has been described by Dr. Martin, and it is a good case to give the profession a precedent, where such results ensue, and there is no apparent cause for them.

*Dr. Jackson* stated that in his opinion necrosis could not be avoided in some cases. He had a case of necrosis occurring in the superior maxillary, where the tooth had been out a number of years. The patient, while working in a wheat field in the summer, suffered severe pain in the head, which after awhile went into the upper left jaw; that there were no teeth there, the patient having worn a plate for some years, and there was no irritation from the plate; there seemed to be a little settling of the muscles, and then immediately came a swelling of the jaw; the third day, a little pus formed; it was lanced freely and the pus ceased, and an injection of iodine, carbolic acid, and glycerine was made, and some cotton put in; as it was on the upper jaw it would free itself more easily than on the lower jaw. In the course of two weeks the sequestra came from it, a large scale was formed nearly an inch long, and about half an inch wide, and it healed very kindly. In this case there had been no injury from any cause whatever. It apparently started as if he had been attack by sun-stroke.

*Dr. Young* said he had a case last summer similar to that of Dr. Martin; a physician asked him to see a patient who seemed to be suffering from alveolar abscess; all his lower teeth on the right side, clear to the median line were affected; they were so loose that they could be tipped either way with the pliers, and the gums were very soft and flabby. At the solicitation of the patient the speaker took out the third molar, which was elongated, and it was hard for the patient to close his mouth; the patient wished the rest taken out, but that request was refused at that time. The patient said that four days before he was working on a farm, and his right lower central incisor seemed to be elongated and sore, and he applied to a dentist, and the dentist made an opening, drilling at the top of the tooth into the nerve-chamber, opening it up and putting in a dressing; that he was to go back to the dentist on Sunday and have it filled, which he did, and on the next day he suffered intense pain in that tooth, and went back to the dentist and wanted it out, the dentist refused to take it out then and gave him some medicine to take, and finally in two days, the tooth being no better, it was drawn; that it seemed to come quite hard; that when he, (the speaker) saw the patient the teeth on the right side were all loose; the day after he first saw him the pus was oozing out around the teeth, and by urgent request all of the teeth on that side up to the cuspid were taken out. The next day the inflammation had extended to the canine on the left side, and the incisors were looser. He wished to have them drawn, but that was not done, as the sacrifice was thought to be too great. The physician wished to have the case in his own hands and the speaker gave him some suggestions as to the treatment, which were followed, but did no good, and the speaker was called in again and found that the inflammation had extended to the second molar on the left side, and in the meantime the canine on the right side had lopped over, so it could be picked out with the fingers; and the day after, the inflammation had extended further, to the third molar on the left side. The patient was then very much emaciated and wished to have all his teeth on the lower jaw taken out, and the physician and the patient were both so urgent, that all those teeth were extracted, several of them being simply taken out with the pliers, after all the teeth were extracted the patient got well at once.

*Dr. Harroun* stated, that in his opinion, a large majority of these cases are caused by contusion; the probability is, that the case of the boy, so ably described by Dr. Martin, was caused by contusion, in biting something harder than usual, which in turn produced inflammation; he (Dr. Harroun) had had some experience in his own mouth as to the effects of contusion, having had three cases of inflammation from that cause, and having lost one tooth after another. In one case he had removed twenty-seven pieces of necrosed bone from the jaw of a patient, making an incision the entire length of the jaw, from one end of the superior maxilla—all starting from contusion in extracting a tooth with undue force—and the pus seemed to be leaking out of every pore in the mouth.

*Dr. Cleland* said that a few days ago he had a case of a young woman who suffered from abscess caused by dead nerve; the swelling was very persistent, and a physician was called in, who made an incision, and the pus that exuded was very offensive. Afterward he (Dr. Cleland), after working in the tooth, cleaned the bone thoroughly and made an application of a solution of nitrate of silver, and the swelling went down. If that case had been allowed to go on without treatment, it would have resulted similarly to that of the boy produced by Dr. Martin.

*Dr. Crawford* said that a strong healthy German came to his office and wanted to have the lower front central incisor extracted, as it ached. It was a sound tooth, and none of his teeth except the second molar slightly, and the third molar considerably, were decayed. The man insisted that the incisor should come out, and after a few days he (the speaker), finally extracted it. The next day there was evidence of extensive inflammation on the left side, and on the third day after the extraction the inflammation had extended the whole length of the jaw. In about a week the two molars were extracted, but it was too late. Necrosis took place the whole length of that lower maxilla, and in about six months the pieces of bone sloughed out, and all his other teeth were loose, but they have since tightened, and they are sound teeth, and the jaw is in good condition; the fistulous opening was probed and the pieces of bone taken out. The man said his lower jaw was broken in extracting that central incisor, and blamed the Doctor for it. What produced the disease? There was not decay enough to produce any trouble. The man worked in a stable and was dirty and indolent. Was the disease caused by his surroundings?

*Dr. Martin* said that bad hygienic conditions no doubt predisposed to that trouble, in the way of poor living, poor food, poor air. It is difficult to trace the cause, and unfair to blame dentists. Dentists have to extract teeth, and everything that is proper may be done and yet necrosis may follow. The prick of a pin may cause serious trouble with some, and yet in thousands of other cases may cause no trouble at all. Dentists should do their duty—first preparing themselves for their work, and then using their best judgment, and if bad results come they cannot help it.

*Dr. Shattuck* stated that that might be all right among the medical and dental professions, but when it gets among the legal profession, the thing is to make the judge and jury believe that duty has been performed; and he was glad that this case of Dr. Martin had been so fully reported, as it was something to show the judge and jury as a precedent.

Discussion upon the paper of Dr. Younger, on "Transplantation of Teeth into Artificial Sockets," after the paper was read by Dr. Metcalf.

*Dr. Allport*, of Chicago, said that although the statements in the paper of Dr. Younger were startling, yet Dr. Younger was a thoroughly honest and intelligent man, and considering his reputation and character, those statements must be taken as facts. Then if we accept the facts, the only question left is as to the process which takes place after the drilling or the making of these artificial sockets. While the speaker had not transplanted teeth from one mouth to another mouth, yet he had seen such cases and knew that it could be done successfully; he himself had replanted a tooth from one position to another position in the same mouth. There is very little difference between the process in the organization of bone around the root of a tooth and the organization of bone between two portions of bone, where the bones come together, and there is space between; the material must be thrown out through the bone, and if that takes place, there is a vacuum, and nature always abhors a vacuum, and something is thrown out, which becomes a nucleus around which the osseous structure is formed. As to drilling the sockets, after the bone has become absorbed, the width of the alveolus must be much narrower than it was before, and it would seem that the roots of the tooth would be long and it would be difficult to get the lateral support, labial and lingual, in many cases, but not anteriorily and posteriorily. In such cases the roots must be shaped to correspond with the width at that point. But while transplantation may be accomplished in some cases successfully, it cannot be made general; in many subjects the soft tissues would not form well, and there might be many forms of disease which might be transmitted; when transplantation is attempted at all it should be confined to thoroughly healthy people.

*Dr. Harroun* said that he knew Dr. Younger to be a man of great skill and great enthusiasm, and from his character it could be concluded that the paper was not written for sensational purposes, but to give to the profession the facts that have come from his experience.

*Dr. Field* stated that he had treated a young lady who lived part of the time in St. Louis and part of the time in Detroit. About six years ago she was brought into his office by her sister to have her right central incisor examined, which was so loose that it could be picked out with the fingers. The doctor said to the young lady that it was broken off directly below the margin of the gum, probably by a severe blow. She denied this, and said it had been getting loose for a good while. She was a nervous, sensitive young lady and would not have the tooth removed and a small plate put in. After that she went to St. Louis, and while she was there, the right cuspid, which had never presented itself in the mouth at all, presented itself directly through the root of the right central incisor, above the margin of the gum. She consulted Dr. Park and Dr. Spaulding, in St. Louis, and they advised the removal of this cuspid and thought there would be enough bone deposited in the socket to make that central incisor fast again. Dr. Spaulding wrote the speaker a long letter about it, and the cause of the tooth being loose was thus made apparent—the cuspid ploughing its way through that root, not destroying the pulp, and nutrition going on as before. Drs. Park and Spaulding made an effort to extract that tooth, but could not get at it, but broke a little part of a cusp. After a time they got at the cuspid tooth, but the central incisor still hung loosely. She afterward consulted Dr. Morrison, of St. Louis, and he said there was a cuspid below which was inside of the arch, on the right side, and he said he would take out the tooth and bore up in the socket of the alveolar process and take out the cuspid below and put it into the place of it. The young lady was averse to wearing an artificial denture, and was willing and brave enough to stand anything, although her temperament was very nervous. Dr. Morrison bored up through the alveolar process with a large burr and took out the cuspid tooth below, and he found the cavity was not large enough, but he made room for that root. After he got the hole bored high enough he cut off the cusp of the tooth and cut that out so he could put a filling in, and he put in a platinum and gold, so as to shape it in the form of a central incisor as much as possible. It is fastened there and is as firm as any tooth in her head, and it requires the most critical examination to tell the difference. The speaker was obliged to confess that at the time he did not think it could be accomplished, but it proved a very successful piece of work. It was heroic treatment.

*Professor Taft* stated that he seen the case described by Dr. Field, that the transplanted incisor (which used to be a cuspid) is as firm in its position as its neighbor and looks as well, and there was no irritation about the gum. That seeing the case described by Dr. Field, prepared the speaker for a better acceptance of the statements made by Dr. Younger in his paper. The truth is that a socket may be formed in the bone and that bone may be so deposited about it as to take hold of that root and fix it in place and render it firm, admits of no doubt. The same thing is done in changing a tooth from one socket to another, whether it is from one person to another or not. But to take out from one socket and put in another socket, where the socket is too small.

one of two things must be done—the root dressed down or the socket burred out, and with both of these methods the teeth have remained fixed in place. The question is presented in the paper as to whether if the cement around the root be taken off, the attachment would be made. That would be an interesting problem, to be decided by experiment. Material would be thrown out in the bony walls in the cavity and adapted to the root and made to take hold upon it and attach to it. There is nothing inconsistent in the statements of Dr. Younger. Take the formation and development of the teeth. The crowns of the teeth before they are erupted are in cavities much larger than the crowns, and if there was not a great deal of filling up when the roots come to be formed, they would rattle around. Bone material is thrown from the walls abundantly and may be formed to and take hold of the root of the tooth. It is doubtful whether any histologist knows exactly the nature of the process that takes place in the genesis of the roots of the teeth. The periosteum is formed upon the root as it grows, necessarily, having a germ for that upon the periphery of the tooth germ; bone matter is thrown up, and makes this attachment. And it is an interesting question to ascertain whether the cavity in which the crown rests has any periosteum at all when it is in that condition. If it has not, the question of a dual membrane becomes exceedingly interesting and one of considerable doubt. That subject was discussed by Prof. Ingersoll, of Keokuk, Iowa, at the last meeting of the American Dental Association. Another factor should be considered: There is great difference in the facility with which living tissue, when it becomes devitalized, will run into decomposition. Some tissues will maintain comparative structural integrity for a comparatively long time. There is very great difference in the tissues of different individuals. The tissues of some persons seem to have a tendency to disintegration and to a return of the elements to their original condition. And this is true with regard to the teeth. Some teeth will become devitalized and assume quickly a yellow or muddy color, and in other cases a tooth may be devitalized for years without any appreciable change of color. This change is dependent mainly upon the decomposition of the organic constituents of the tooth substance, of the dentine itself; and after some teeth are removed we sometimes find the organic structure maintains its integrity, without a tendency to decomposition. If like conditions and surroundings are brought to it, it can readily be conceived that a living tissue that was acceptable to it would take hold of and unite with it; but if a tooth has undergone decomposition in its organic portions, it would be useless to place such a tooth in a socket anywhere. It is important, therefore, in transplantation, to have a tooth which, although the organic portion has lost its moisture, yet maintains its organic structure and is in a condition to invite, or at least accept, the living material that may be brought in contact with it under the vital influences surrounding it. It is important that the teeth to be used should be placed in conditions that will retard decomposition, hence the reasonableness of placing them in a living tissue, as in the comb of a cock, or in water of about blood-heat temperature and keeping them there, so as to keep them free from infection. Sometimes, in the transplantation of teeth, they have been taken out and permitted to remain and undergo decomposition, which would render it impracticable that the union could be effected."

After the reading of the paper of Dr. Land, upon "Adhesive Dentures," the paper was discussed by Drs. Watling, Dorrance, Field, Jackson, and Land.

